# Production traits in F_1_ and F_2_ crosses with naturalized hair breed Santa Inês ewes

**DOI:** 10.1186/2193-1801-3-66

**Published:** 2014-02-03

**Authors:** Alessandra Ferreira da Silva, Concepta McManus, Tiago do Prado Paim, Bruno Stéfano Lima Dallago, Geisa Isilda Ferreira Esteves, Helder Louvandini, José Braccini Neto, Carolina Madeira Lucci

**Affiliations:** Faculdade de Agronomia e Veterinária, Universidade de Brasília, Brasília, Distrito Federal 70910-900 Brazil; INCT-Pecuária, Brasília, Distrito Federal 70910-900 Brazil; Departamento de Zootecnia, Universidade Federal do Rio Grande do Sul, Avenida Bento Gonçalves 7712, Porto Alegre Rio Grande do Sul, 91540-000 Brazil; Centro de Energia Nuclear na Agricultura, Universidade de São Paulo, SP CP 96, Piracicaba, CEP 13.400-970 Brazil; Departamento de Ciências Fisiológicas, Instituto de Biologia-UnB, Universidade de Brasília, Campus Brasília, Brasília, DF CEP: 709010-90 Brazil

**Keywords:** Sheep production, Milk, Females, Performance, Fertility, Genetic resource

## Abstract

The once bred ewe slaughter method proposes the use of female lamb to produce a lamb and then both are slaughtered, increasing income and high quality meat production. Thus, this study evaluated the growth and reproduction performance of ewe lamb from Santa Inês (SI), a naturalized genetic resource, and their crosses (Dorper x Santa Inês (DOR), Texel x Santa Inês (TEX), Ile de France x Santa Inês (ILE)), as well as the survivability and development of their offspring. The animals were weighed monthly from birth to 12-months age. Samples of milk were collected on approximately 30 days of lactation. The physical-chemical analysis of milk was performed. SI females (2.94 kg) had significantly lower birth weight than DOR (3.80 kg) and TEX (3.87 kg). ILE females had higher weaning weight and weight at 12 months than SI females, which reflected in higher daily weight gain (ADG) (108.46 g/day) than TEX and SI. The pregnancy rates at 12 months were ILE (57.14%), TEX (25%), DOR (50%), and SI (28.57%), with TEX and SI differing of ILE and DOR (p = 0.03). Therefore, in semi-confinement and in a once-bred ewe production system using crossbreeding and allying meat production and reproduction, we recommend the use of Dorper and Ile de France breeds for crossbreeding with Santa Inês females. These results demonstrated the useful of a local genetic resource in productive system aiming a low cost meat production.

## Introduction

The sheep flock in the Center-west of Brazil is used mainly for meat production. The Santa Ines hair breed, a naturalised hair breed, is the most popular in this region as it exhibits estrus throughout the year and has no need for shearing. However, their lambs have a lower rate of weight gain and poorer carcass conformation when compared to specialized meat breeds (Alcalde et al. 2004) and consequently lower carcass yield (Garcia et al. 2010). Its use in crossbreeding programs has been proposed as a maternal breed with specialized breeds for meat production (Paim et al. 2013).

The major source of cost in lamb production is the ewe maintenance (Paim et al. 2011; McManus et al. 2011). One system proposed using modelling to reduce this cost is the once bred ewe slaughter method (Keeling et al. 1991), where the females are mated at 6 to 8 months of age, weaning a lamb at 16 to 18 months of age and then slaughtered before 22 months of age, producing a high-weight carcass without quality losses. In this system, the ewe maintenance cost is reduced as the female still growing while producing lamb.

The lamb survival and early development are directly dependent on maternal lactation activity and has a direct effect on pre-weaning weight gain of lambs (Hassan 1995). In this context, Ferreira et al. (2011) describe that F_1_ females have higher milk production than their maternal breeds due heterosis and complementarity between breeds. However, lactation may be influenced by many factors, such as the paternal genetic group of the dam, the number of lambs born and fed, the season, weight gain of the dam through pregnancy until weaning (Morgan et al. 2006; Morand-Ferr et al. 2007).

Lamb mortality is a challenge for sheep production systems (Paim et al. 2013). Much of the lamb mortality occurs in the first weeks of life (Nowak et al. 2000), where survival depends on the complex interaction between the ability of mother to care for it and lamb viability. Therefore, ewes at the first parturition may have low mother care which can impact the lamb mortality rate.

There is a need for studies evaluating the performance and reproduction parameters of lamb ewes from birth to reproduction at 12 months old and their offspring performance, aiming to support the once bred ewe slaughter system. Thus, this study aimed to evaluate growth and reproductive parameters of ewes from crosses between four paternal breeds with Santa Inês dams reared at semi-intensive system and the impact on survival rate and weight gain of their offspring.

## Material and methods

Sixty six ewe lambs were used, all from Santa Inês dams, a naturalized hair breed, crossed with Santa Inês (SI) (n = 14), Dorper (DOR) (18), Texel (TEX) (20) and Ile de France (ILE) (14) rams. Three different purebred rams of each breed were used. All rams were bought from different sources, they were from different farms and did not contain any common ancestors for at least five generations.

All were born within a month of each other (August – middle of dry season in the region), when they were identified and individually weighed. Fecal egg count (FEC) was measured every 45 days, and deworming carried out when the results were greater than 500. All received concentrate (76% ground corn plus 24% soybean meal) in creep feeding at 100 g/animal/day and had access to *Andropogon gayanus* pasture pre-weaning. Weaning was performed at 90 days of age. Post-weaning, as well as access to pasture, the ewe lambs received concentrate (55% ground corn, 30% soybean meal, 10% cottonseed and 5% wheat meal) at 300 g/animal/day. Mineral salt and water were offered *ad libitum*.

All animals were weighed monthly. Vasectomized rams were used at a ratio of 1:30 for estrus observation. The ewe lambs entered in service after the sixth month of age and two normal estrus behaviors or after eight months of age, irrespective of heat observation. Four Dorper rams, different from the F1 sire, were used for crossing with the F1 crossbred ewe lambs. Diagnosis of pregnancy was carried out on the 45th day after estrus using transabdominal ultrasound.

Three milk samples were collected from these F1 ewes on three occasions between 25 and 45 days of lactation, which corresponds to the peak of lactation according to literature (Souza et al. 2005; Brito et al. 2006; Van der Linden et al. 2009). For each sample, 30 ml of milk were collected using protective screens against possible contamination and immediately refrigerated. The physical-chemical analyses (fat (FAT), nonfat dry matter (NDM), protein (PTN) and lactose (LAC) as well as density (DEN) and cryoscopic point (CRYO)) were performed using the ultrasonic device EKOMILK® (EON Trading PLC, USA).

Data available on both F1 dam and TC lambs included birth weight (BW), weaning weight (WW), and the average daily gain pre weaning (preWG), post weaning (postWG) and mean up to one year of age (ADG). Weaning was performed at 90 days of age. TC lamb feeding was the same as the F1 as described above.

Analysis of variance was performed using PROC MIXED in SAS®, considering a significance level of 5% and using the Tukey test for mean comparison. Birth weight and type of parturition and rearing (single or twins) was considered as fixed effects, as well as, sex in analysis of TC lambs data. Correlations used depended on the type of variable: Kappa correlations for binomial traits (0/1), Spearman for categorical traits and Pearson for quantitative traits. Mortality data and pregnancy rates were analyzed using logistic regression (evaluating odds ratio and contrast tests between genetic groups).

## Results

There was no difference in weight at 12 months for ewes from ILE (40.69 ± 6.90 kg), TEX (33.92 ± 8.80 kg), DOR (37.28 ± 8.30 kg) and Santa Inês ewes (34.23 ± 10.04 kg). The performance and weight results are showed in Table 1.

**Table 1:** **Mean and standard deviations of Birth weight (BW), weight gain pre-weaning (preWG), weaning weight (WW) post weaning weight gain (postWG) and average daily gain to 12 months of age (ADG) in four genetic groups of ewe lambs**

	BW (kg)	preWG (g/day)	WW (kg)	postWG (g/day)	ADG (g/day)
Genetic group					
Santa Inês (SI)	2.94 ± 0.67^b^	85.21 ± 26.08^a^	11.11 ± 3.14^a^	88.79 ± 36.45^a^	90.64 ± 26.27^b^
Ile de France x SI	3.45 ±0.7^ab^	103.3 ± 28.13^a^	14.03 ± 2.62^b^	71.71 ± 29.27^b^	108.16 ± 18.5^a^
Dorper x SI	3.80 ± 0.82^a^	93.83 ± 28.36^a^	13.50 ± 2.71^b^	99.78 ± 24.76^a^	99.86 ± 22.10^ab^
Texel x SI	3.78 ± 0.72^a^	83.20 ± 33.47^a^	11.81 ± 3.66^ab^	82.80 ± 25.12^a^	91.44 ± 22.60^b^
Type of birth					
Single	3.54 ± 0.84^a^	93.43 ± 29.82ª	12.80 ± 3.20^a^	88.43 ± 30.60ª	98.86 ± 23.24ª
Twin	3.33 ± 0.51^a^	89.38 ± 30.42ª	11.73 ± 3.57^a^	74.75 ± 23.58ª	89.73 ± 21.27ª
Mean	3.56 ± 0.40	91.92 ± 26.27	12.70 ± 1.31	84.74 ± 30.90	89.00 ± 22.90

Superscripts with different values in the same column differ significantly using the Tukey test (P <0.05). SI: Santa Inês.

The rates of pregnancies at 12 months were ILE (57.14%), TEX (25%), DOR (50%), and SI (28.57%). The contrast test between TEX and SI versus ILE and DOR showed statistical difference (p = 0.03), but the others contrast tests comparing genetic groups (each pair) did not differentiate genetic groups (p > 0.05).

Despite the similarity in preWG between the genetic groups (Table 1), the ewe that became pregnant showed higher weight gain pre-weaning (108.16 ± 24.54 g/day) than those who did not become pregnant (83.00 ± 28.76 g/day), regardless of genetic group. The post weaning gain did not influence pregnancy rates. The ADG differed between pregnant (102 ± 22.46 g/day) and non-pregnant ewes (86.83 ± 22.45 g/day). The weight at 12 months was higher in the pregnant ewes (39.49 ± 7.82 kg) than non-pregnant ewes (34.29 ± 7.97 kg).

Milk from TEX females had significantly lower values for FAT, PTN, LAC, NDM when compared to crossbred groups and DEN and CRYO when compared to the pure group (Table 2). During pre-weaning, survival rates of 75%, 78%, 75% and 100% for SI, DOR, ILE and TEX ewe lambs, respectively, were observed, with no difference between groups. There were no differences in birth weight, weaning and consequently in daily weight gain between lambs from the maternal distinct genetic groups studied (Table 3). All lambs born to F1 ewes were singletons. The genetic group of dams did not influence the survivability of the lambs.

**Table 2:** **Average values for percentage (%) of fat (FAT), protein (PTN), lactose (LAC) and nonfat dry matter (NDM) for milk from ewes of four genetic groups**

Genetic group	FAT	PTN	LAC	NDM	DEN	CRYO
Santa Inês (SI)	8.25 ± 1.40^ab^	3.72 ± 0.18ª	6.04 ± 0.28^a^	10.51 ± 2.64^a^	34.45 ± 2.71ª	57.24 ± 3.74ª
Ile de France x SI	9.31 ± 3.47^a^	3.58 ± 0.35ª	5.76 ± 0.56^a^	10.11 ± 0.99^a^	32.49 ± 5.20^ab^	54.68 ± 7.92^ab^
Dorper x SI	8.49 ± 3.68^a^	3.70 ± 0.56ª	5.96 ± 0.80^a^	10.47 ± 1.49^a^	33.93 ± 7.07ª	54.40 ± 5.25^ab^
Texel x SI	6.34 ± 3.37^b^	3.18 ± 0.44^b^	5.24 ± 0.60^b^	9.12 ± 1.14^b^	30.30 ± 3.51^b^	51.53 ± 5.80^b^
Mean	8.25 ± 3.20	3.57 ± 0.45	5.78 ± 0.64	10.12 ± 1.19	32.87 ± 5.06	54.61 ± 6.21

Superscripts with different values in the same column differ significantly using the Tukey test (P <0.05). SI: Santa Inês.

**Table 3:** **Birth weight (BW), weaning weight (WW) and average daily gain (ADG) of TC lambs born to dams from four genetic groups**

Dam genetic group	BW (kg)	WW (kg)	ADG (g/day)
Santa Inês	4.18 ± 0.37ª	20.27 ± 5.06ª	177 ± 0.05ª
Ile de France x Santa Inês	4.31 ± 0.88ª	18.48 ± 2.01ª	152 ± 0.03ª
Dorper x Santa Inês	4.16 ± 0.68ª	20.07 ± 2.86ª	175 ± 0.03ª
Texel x Santa Inês	4.17 ± 0.73ª	19.72 ± 3.36ª	172 ± 0.04ª
Mean	4.21 ± 0.69	19.58 ± 3.52	169 ± 0.03

Superscripts with different values in the same column differ significantly using the Tukey test (P <0.05).

Birth weight had low correlation with other weights and weight gains (Table 4) while weaning and 12 month weights had higher correlations between them. The correlations between weight and growth rates in the dams with those of their offspring were also generally low and in some cases negative. Correlations between all milk quality measures were high (Table 5).

**Table 4:** **Correlations between weights and weight gain in four genetic groups of sheep**

	BW	WW	W12	ADG	preWG	postWG	Birth type	Rearing type	BW_TC	WW_TC
WW	0.40									
W12	0.21	0.56								
ADG	0.11	0.50	0.99							
preWG	0.17	0.92	0.59	0.58						
postWG	0.06	0.19	0.92	0.93	0.25					
Birth type^1^	-0.39	-0.23	-0.14	-0.09	-0.11	-0.06				
Weaning type^1^	-0.35	-0.27	-0.06	0.00	-0.12	0.05	0.72			
Pregnancy	-0.06	0.22	0.31	0.32	0.25	0.27	-0.04	-0.11		
BW_TC	-0.10	-0.27	-0.31	-0.32	-0.29	0.08	0.43	0.55		
WW_TC	-0.10	0.26	0.15	0.15	0.12	-0.04	0.36	0.35	-0.16	
ADG_TC	0.00	0.26	0.21	0.21	0.21	0.00	-0.20	-0.30	-0.29	0.95

BW – birth weight; WW – weaning weight; W12 –weight at 12 months of age; preWG – preweaning weight gain; postWG – post weaning weight gain; ADG – average daily gain from birth to 12 months; TC – threecross lambs. ^1^Refer to birth and weaning type of dam.

**Table 5:** **Correlations between milk quality measures from ewe lambs at 30 days of lactation**

	Fat (%)	Dry matter (%)	Density (g/mL)	Protein (%)	Cryoscopy index (oH)
Dry matter	0.02				
Density	-0.41	0.85			
Protein	0.08	1.00	0.81		
Cryoscopy index	-0.67	0.67	0.84	0.63	
Lactose	-0.03	1.00	0.87	0.99	0.70

## Discussion

The mean birth weight is directly related to genetics and nutrition of pregnant sheep, while weaning weight depends mainly on milk production and the availability of solid foods to lambs (Pires et al. 2000). The highest birth weight group was for DOR which differed from SI, but did not differ from ILE and TEX, showing the possible effect of heterosis on this trait. Observations of interaction between maternal and paternal genotype for birth weight have been previously described (Freking and Leymaster 2004).

There are no significant differences between groups for preWG. The SI dams may have influenced weight gain during this period. According to Quesada et al. (2002) and Matika et al. (2003) several sheep breeds have shown that maternal influences are important for birth weight and weaning.

There was no significant difference in weaning weight between TEX, ILE and DOR. However, ILE and DOR had higher WW than SI, demonstrating a heterosis effect in these two groups. These higher WW probably was related to higher BW in the first groups, whereas there was no difference between genetic groups in preWG. Moreover, these results demonstrated that, despite the higher BW of TEX females compared to SI, this was not maintained at weaning. This was also seen by Villarroel et al. (2006) with TEX and SI lambs and can be explained by the fact that milk production from dam influences directly the weaning weight (Motta et al. 2000; Villarroel et al. 2006). It is possible that the milk produced by Santa Inês ewes may have been insufficient for Texel cross lambs to express their full genetic potential for growth as a specialized meat breed.

The postWG was lower in ILE compared to DOR. In a similar study, Paim et al. (2013) also found a low post weaning gain of Ile de France cross lambs comparing the same genetic groups. These results may be associated with a lower adaptability of ILE to conditions of the experiment such as low quality tropical pastures, low humidity and high daily temperatures. However, no difference on pregnancy rates between genetic groups was seen. No difference in postWG was observed between pregnant ewe lambs (86.87 ± 32.50 g/day) and non-pregnant (86.48 ± 29.26 g/day). This demonstrates that performance post weaning did not impair the reproduction traits.

On the other hand, good performance in pre-weaning weight is critical to achieve better fertility rates in the future, since pregnant ewe lambs put on more weight pre-weaning compared to not pregnant. Independent of genetic group, the ewes diagnosed positive for pregnancy had higher preWG (103.96 ± 23.36 g/day) than those not pregnant (93.57 ± 22.46 g/day). Therefore, these results highlight the impact of ewe performance during the first months of life in their further reproductive parameters.

The daily average weight gains (ADG) were similar between DOR, SI and TEX. The offspring from specialized meat breeds are expected to have a higher growth rate than naturalized breeds, nevertheless this was not seen. It can be a result of the crossbreeding suffered by SI in recent years to increase mature size and growth rates (McManus et al. 2010). Moreover, this result demonstrated the good potential for meat production of SI. Similar to this study, Garcia et al. (2010) observed no difference in ADG between SI (107 g/day), TEX (111 g/day) and DOR (107 g/day), in confinement.

ILE females (44.14 ± 6.90 kg) had higher mean weight at 12 months when compared to SI (37.04 ± 10.04 kg). This may be due to the superior mature weights of Ile de France breed compared to Santa Inês, and it can impact the production cost if these ILE ewes are maintained in breeding flock. On the other hand, in a once bred female system as proposed here, the higher ewe weight represents a higher income at the slaughter of this ewe.

The TEX and SI ewes showed a lower pregnancy rate than ILE and DOR. Therefore, the heterosis effect and complementarity between breeds showed significant effect in ILE and DOR, improving the results found in pure Santa Inês ewes. Hence, these crosses may proportionate higher incomes to the system due to higher lamb production in a shorter time.

The nonfat dry matter comprises a solid portion of milk excluding lipids. TEX also had the lowest values (9.12%) when compared to the other groups. So, Texel crosses ewes had lower solid proportions in milk compared to other genetic groups.

According to Park et al. (2007), the average concentration of protein in sheep milk is 5.8%. However, this value may vary according to genetic group, stage of lactation, feeding, climate, season and udder health status. Here, 3.57% was the average of protein observed considering all genetic groups. The reduced amount of protein observed in this experiment may be associated with the physiological condition of primiparous ewes, since according Sevi et al. (2000), milk quality improves with advancing age and lactation. This was also reported by Economides (1986) who noted that higher concentrations of nutrients in multiparous compared with primiparous Chios ewes.

According to Hassan (1995) the main factor that influences lamb weight gain in pre-weaning is the mother’s milk. However, despite the observation of different chemical compositions in genetic groups, there was no influence of this condition on the performance of weight gain of their lambs during lactation.

The levels of glucose, a structural component of lactose may influence the growth of lambs (Wilson et al. 1983). However, low levels of lactose observed in the milk of TEX did not influence the pre-weaning performance of their lambs compared to the other groups. Similarly, Peeters et al. (1992) reported that the composition of milk from three genetic groups (Flemish milksheep, Suffolk and Texel) did not affect the weight gain of their lambs.

## Conclusions

Female Santa Inês and Texel x Santa Inês had grown slower than Ile de France x Santa Inês, in semi-confinement. Ewe lambs from Dorper and Ile de France crosses with Santa Inês dams showed the best performance at weaning and maintained this advantage post weaning, and also had the best pregnancy rates at 12 months of age. Lower values of fat, nonfat dry extract, protein and lactose were observed in TEX. Despite this, there was no difference in weight gain in the first months of life of their offspring, which demonstrates a low energy loss of these females that may proportionate productive advantage in a nutrition-restricted environment. Dorper and Ile de France crossbred with Santa Inês females are recommended to once bred ewe production system. The results of this study highlight a once bred ewe production system that can increase sheep meat production using a local genetic resource.

### Ethics committee

Approved by the ethics committee for animal use of Universidade de Brasília, process n° 33/2009.
